# Impact of interleukin-6 promoter polymorphism and serum interleukin-6 level on the acute inflammation and neovascularization stages of patients with Eales’ disease

**Published:** 2011-10-01

**Authors:** Aditi Sen, Suman Kalyan Paine, Imran Hussain Chowdhury, Amrita Mukherjee, Subhadip Choudhuri, Avijit Saha, Lakshmi Kanta Mandal, Basudev Bhattacharya

**Affiliations:** 1 Department of Biochemistry, Dr. B C Roy Post Graduate Institute of Basic Medical Education and Research (IPGME&R), Kolkata, India; 2 Regional Institute of Ophthalmology, Kolkata, India

## Abstract

**Purpose:**

To evaluate the role of interleukin-6 (IL-6) in the inflammatory and proliferative stages of Eales’ disease (ED) and to determine the influence of IL-6–174G/C polymorphism in the IL-6 and IL-6-regulated protein expression, as well as the development of ED.

**Methods:**

One hundred and twenty-one patients diagnosed with ED, 223 matched healthy controls, and 16 control patients with macular holes were recruited from the eastern Indian population. Serum and vitreous levels of IL-6 and vascular endothelial growth factors (VEGF) were measured by enzyme-linked immunosorbent assay. Serum levels of high-sensitivity C-reactive protein (hsCRP) were measured by enzyme immunoassay. Subjects were genotyped for the IL-6–174G/C polymorphism (rs1800795) by a custom TaqMan single-nucleotide polymorphism (SNP) Genotyping Assays system.

**Results:**

Serum IL-6 (p<0.0001), hsCRP (p<0.0001), and VEGF (p=0.0031) levels were significantly higher in the inflammatory stage of ED than in healthy controls. Serum IL-6 also significantly correlated with hsCRP (Spearman’s correlation coefficient; r=0.4992, p=0.0009), but not with VEGF in this stage in ED patients. At the proliferative stage of ED, significantly higher levels of vitreous IL-6 (p=<0.0001) and VEGF (p=<0.0001) were found compared with the vitreous of patients with macular holes. A significant correlation was observed between vitreous IL-6 and VEGF in ED patients (Spearman’s correlation coefficient; r=0.5834, p=0.0087). A statistically significant association was found between the −174GG genotype (p=0.006) and occurrence of ED. Mean serum and vitreous concentrations of IL-6 were also higher in the subjects with the GG genotype than in those with the GC or CC genotype in this population.

**Conclusions:**

IL-6 expression, regulated by the allelic distribution of −174 loci and the enhanced level of IL-6, modulates CRP and VEGF concentration depending respectively on the acute inflammatory stimulation at the initial stage and angiogenic stimulation at the advanced stage of ED.

## Introduction

Eales’ disease (ED) is an idiopathic inflammatory vasoproliferative disease of the retina primarily affecting the peripheral retina of individuals in the third and fourth decade of life [1,2]. It is predominantly found in the Indian subcontinent [3]. The etiopathogenesis of this disease still remains an open experimental issue, but pathologically it is characterized by retinal perivasculitis mainly affecting the peripheral retina (inflammatory stage), leading to sclerosis of the retinal vessels indicating retinal ischemia (ischemic stage), and finally retinal neovascularization, recurrent vitreous hemorrhage, with or without retinal detachment (proliferative stage) [3,4]. The association of human leukocyte antigen [5] and T-cell involvement in the lymphocytic infiltration in the epiretinal membrane of patients with ED [6] indicate that the T-cell-mediated immune mechanism might play a key role in retinal vasculitis i.e., the inflammatory stage of this disease.

Interleukin-6 (IL-6) is a multifunctional cytokine with a proinflammatory character, and is thought to be one of the major mediators in driving the acute phase immune response [7]. IL-6, produced by cells of the innate and adaptive immune system, induces B-cell growth and differentiation [8], and act as an early mediator of acute phase inflammatory proteins, such as C-reactive protein (CRP) expression [9,10] and contributes to the activation and /or proliferation of T cells [9,11]. Several studies have suggested that assessments of IL-6 and CRP are valuable tools for predicting systemic inflammation in different pathologic conditions [12,13]; these two proteins were also elevated in ED pathogenesis [14-16].

IL-6 is also considered to be an indirect inducer of angiogenesis through the induction of vascular endothelial growth factor (VEGF) [17,18], a potent angiogenic factor [19] involved in several pathological angiogenesis in the retina including ED [15,20].

The effect of cytokines generated during the inflammatory stage of ED [14] clearly indicates their involvement in the proliferative stage, i.e., the severity of this disease and variation in cytokine production in all probability affects the extent and severity of the disease. As the magnitude of cytokine production does not depend only on antigenic challenge but also on host genetic factors [21], the search for single-nucleotide polymorphism (SNP) has now become a potential tool not only for better understanding of the etiopathogenesis of the disease, but also as a probable marker of disease susceptibility and severity. It has been demonstrated that IL-6 promoter polymorphisms are key regulators of IL-6 gene and downstream protein levels in vitro and in vivo [22]. Polymorphism of position −174 is one of the several IL-6 polymorphisms that have been suggested to affect IL-6 expression [23], and has been investigated in a wide variety of diseases. Therefore, this polymorphism may be predisposing factor for the development of ED in an individual.

This study was conducted to investigate whether IL-6 acts as a modulator in the inflammatory and proliferative stages of ED by regulating expression of acute phase inflammatory protein and potent angiogenic factor, respectively, as well as to determine the influence of IL-6–174G/C polymorphism in the IL-6 and/or IL-6-regulated protein expression and the development of ED.

## Methods

### Study subjects

One hundred and twenty-one patients (97 males, 24 female) with ocular findings suggestive of ED, representing different stages of the disease, were recruited from a retina research clinic at the Regional Institute of Ophthalmology, Kolkata, India between 2007 and 2010. Forty-one patients presented with active vasculitis in the periphery of the retina, 50 patients had neovascularization in the periphery, and 30 patients had developed an advanced stage of neovascularization in the disc and periphery with vitreous hemorrhage and tractional retinal detachment.

Ophthalmological diagnosis was performed for all patients by dilated fundus examination with direct and indirect ophthalmoscopy, slit lamp biomicroscopy with +90D and 3-mirror lens, stereoscopic color fundus photography and fluorescein angiography. Eyes exhibiting vitreous hemorrhage underwent ultrasonography to detect tractional retinal detachment. Other detailed ophthalmic examinations included visual acuity determination by Early Treatment Diabetic Retinopathy Study (ETDRS) chart, intraocular pressure measurement by applanation tonometry, and anterior segment evaluation by slit lamp examination. The location and extent of retinal involvement by vasculitis, nonperfusion, neovascularization, and fibrovascular traction were documented in all patients by digital color fundus photography and fluorescein angiography.

As ED is characterized by retinal phlebitis, peripheral nonperfusion, and retinal neovascularization, fundus examination reveals a different clinical picture according to the stage of disease progression that patients present.

In relation to the inflammatory stage, phlebitis was manifested in the patients of our study as venous dilation with tortuosity and discontinuity of veins with flame shaped hemorrhages and perivascular exudates along peripheral veins. Vascular sheathing was found in some patients in a few or more vessels ranging from continuous thin white lines to segmental heavy exudation. The number of patients in the inflammatory stage was low in this study due to less attendance of these patients to the hospital, as they are usually asymptomatic regarding loss of vision, which is a subjective phenomenon. Moreover, patients usually report when they have a marked loss of vision due to vitreous hemorrhage from new blood vessels or in the advanced proliferative stage when they have lost perception of light. Some patients in the proliferative stage of ED present neovascularization of the disc, whereas some exhibit neovascularization elsewhere, located at the junction of perfused and nonperfused retina. In others, where vitreous hemorrhage has already occurred, fundus details are usually obscured, but old vitreous bleed leading to fibrous organization, retinitis proliferans, and subsequently tractional retinal detachment may be seen.

Among those patients who were in the proliferative stage (80 patients), 19 of them had undergone pars plana vitrectomy and undiluted vitreous was collected from these patients for selective protein study.

Sixteen patients (15 males, 1 female) who underwent vitrectomy and internal tamponade for idiopathic macular holes were included in this study as control for vitreous study. This disorder is caused by vitreomacular traction occurring before posterior vitreous detachment and there are no signs of ischemia, proliferation, or inflammation.

Two hundred twenty three (183 males, 40 females) age- and sex-matched healthy adults, without any history suggestive of ED, attending the outpatient department of the same institute for treatment of visual difficulty due to refractive errors without any other systemic or ocular pathology. provided the blood samples and constituted our healthy control group.

Subjects with a history of diabetes mellitus, hypertension, collagen vascular disease, HIV, symptomatic arthritis, symptomatic malignancy, sarcoidosis, Behcet’s disease, systemic lupus erythematosus, Coats’ disease, and syphilis were excluded from the study. The study protocol according to the declaration of Helsinki was approved by Institution’s Ethical Committee and informed consent was obtained from each subject.

### Sample collection and preparation for analysis

Ten milliliters of whole blood from ED patients and healthy controls were collected by venipuncture from peripheral veins. Seven milliliters of blood were taken in an EDTA-containing tube for peripheral blood mononuclear cell layer separation. The other 3 ml were collected in a clot vial and allowed to clot. After clot retraction, serum was separated by centrifugation at 3,000× g at 4 °C for 20 min and was frozen at −80 °C until further use.

Vitreous samples were collected from the study subjects and control patients who underwent three-port pars plana vitrectomy. After the construction of the ports, a vitreous cutter was introduced in the mid-vitreous; before turning the infusion fluid, 200 μl of undiluted vitreous gel was excised and aspirated into the handheld sterile syringe attached to the suction port of the vitrectomy probe, using manual suction with a high cutting rate. The vitreous biopsy samples thus obtained were immediately put in ice and centrifuged at 10,000× g for 15 min at 4 °C. After centrifugation, supernatants were divided into two equal aliquots and stored at −20 °C for immediate use or at −80 °C for future use. Simultaneously venous blood was collected from the study subjects and control patients, who underwent vitrectomy, at the time of vitrectomy and processed as stated above.

### Genomic DNA isolation and genotyping of interleukin-6–174G/C (rs1800795) polymorphism

Genomic DNA was isolated from peripheral blood mononuclear cells by proteinase-K digestion and the standard high salt extraction method [24]. Genotyping of the −174G/C polymorphism in the 5′ regulatory region of the IL-6 gene in the patient and healthy control groups was performed by a custom TaqMan SNP Genotyping Assay system on the 7300 ABI Real-Time PCR system (both from Applied Biosystems, Foster City, CA) using 96 well plates. The sequences of forward and reverse primers and two probes for the −174C and −174G alleles are listed in Table 1.

**Table 1:** Sequences of primers and probes used for *IL-6–174G/C* genotyping

**Primer and probe name**	**Sequence**
Forward primer	5′-CGACCTAAGCTGCACTTTTCC-3‘
Reverse primer	5′-GGGCTGATTGGAAACCTTATTAAGATTG-3′
C-^174 allele^ probe	VIC-CCTTTAGCATgGCAAGAC
G-^174 allele^ probe	FAM-CCTTTAGCATcGCAAGAC

### Measurement of interleukin-6, high-sensitivity C-reactive protein, and vascular endothelial growth factor concentration

Total concentrations of IL-6 and VEGF in the serum and vitreous of study subjects were measured using a commercial enzyme-linked immunosorbent assay kit (R&D System, Inc., Minneapolis, MN), with a minimal concentration detection limit of 0.039 pg/ml and 5.0 pg/ml, respectively, according to the manufacturer’s instructions. Serum hsCRP concentrations were measured using the UBI Magiwel Enzyme Immunoassay (United Biotech Inc., Mountain View, CA) as per the manufacturer’s instructions. The assay is reported to have a minimum detectable concentration of 0.00035 mg/l.

### Vitreous protein estimation

Vitreous protein (mg/ml) levels were estimated by the bicinchoninic acid protein assay method [25] using bicinchoninic acid reagent (Sigma-Aldrich, St. Louis, MO) containing 4% copper sulfate.

### Statistical analysis

Age and sex differences between the patients and controls were investigated by the Student *t* test and chi-square (χ^2^) test, respectively. The significance of difference of protein concentration between corresponding groups of observations was evaluated by the Mann–Whitney U test and all values were expressed as mean (standard deviation) and range. The correlations between study parameters were analyzed by Spearman’s correlation test (by GraphPad Software). The study groups were tested for the Hardy–Weinberg equilibrium and expected and observed frequencies were compared by χ^2^ analysis (by an online calculator: provided by Tufts University, Boston, MA). The allele and genotype frequencies for each SNP were compared between ED patients and controls using the χ^2^ analysis on a 2×2 contingency table and calculation of the odds ratio with a 95% confidence interval. Fischer’s exact p values were calculated for analysis when one or more variables within the 2×2 tables were less than 5 (by online calculator provided in the public domain by Vassar College, Poughkeepsie, NY). Power calculation was conducted using PS Power and Sample Size Calculation software version 3.0.34. The Bonferroni correction of the significance level was applied to account for multiple testing. The level of statistical significance was set at p<0.05, except for tests in which Bonferroni adjustment was applied.

## Results

The demographic and clinical characteristics of the study participants are presented in Table 2. There was no significant difference in age (p=0.1424), sex (p=0.662), ethnicity, or geographic origin between the ED patients and healthy controls whose SNP was analyzed as a whole.

**Table 2:** Demographic and clinical characteristics of the study participants

**Characteristics**	**ED patients (n=121)**	**Controls (n=223)**	**Control patients with macular hole (n=16)**
Males: Females	97:24	183:40	15:1
Mean age [years (SD)]	30.6 (10.7)	32.26 (9.6)	61.2 (7.5)
Type of lesion			
Inflammatory stage			
Active vasculitis in peripheral retina	41	-	-
Proliferative stage			
Neovascularization in the peripheral discs	50	-	-
Vitreous hemorrhage with / without tractional retinal detachment	30	-	16
Visual acuity			
20/20 −20/40	38	212	-
20/50 – 20/70	23	11	-
20/80 – 20/100	30	-	6
20/200 – 20/400	30	-	10

### Serum level of interleukin-6, high-sensitivity C-reactive protein, and vascular endothelial growth factor, as well as their correlation in patients with Eales’ disease in the inflammatory stage

Table 3 summarizes the demographic and laboratory measurements of IL-6, hsCRP, and VEGF in ED patients in the inflammatory stage of the disease (n=41) and compared the values to those of the healthy controls (n=223). There was no significant difference in age (p=0.09) or sex (p=0.1750) between the selected group of patients and healthy controls.

**Table 3:** Demographic and laboratories characteristics of the Eales disease (ED) patients in inflammatory stage and healthy controls

**Characteristics**	**ED patients (inflammatory stage; n=41)**	**Healthy controls (n=223)**	**p value**
Males: Females	29:12	183:40	0.09
Mean age [years (SD)]	30.1 (7.8)	32.26 (9.6)	0.175
Serum IL-6 (pg/ml)	27.21 (19.06)	4.795 (2.669)	<0.0001*
Serum hsCRP (mg/l)	2.676 (0.6142)	0.9343(0.1455)	<0.0001*
Serum VEGF (pg/ml)	308.8 (73.42)	265.3 (79.65)	0.0031*

Values are mean (SD) * Significant P value

The mean serum concentration of IL-6 was higher in the inflammatory stage in ED patients compared to the healthy controls (27.21 pg/ml [7.56–78.12 pg/ml] versus 4.795 pg/ml [1.04−10.56 pg/ml]; p<0.0001). Similar trends were also observed for hsCRP (2.676 mg/l [1.670–3.987 mg/l] versus 0.9343 mg/l [0.56–1.43 mg/l]; p≤0.0001) and VEGF (308.8 pg/ml [136.3–410.4 pg/ml] versus 265.3 pg/ml (123.7–368.4 pg/ml]; p=0.0031).

**Figure 1:**
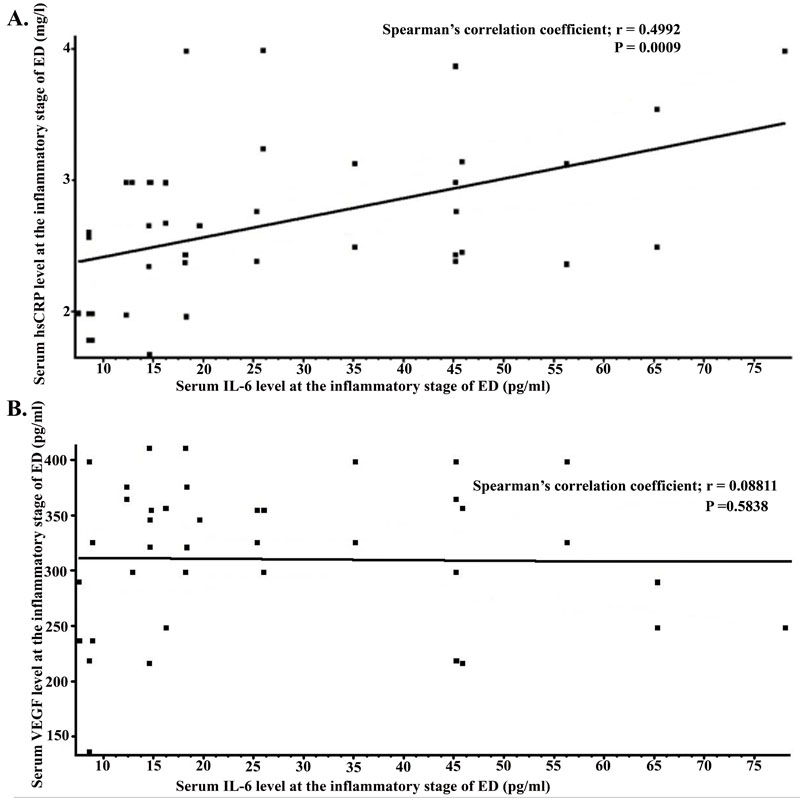
Relation between interleukin (IL)-6 and high-sensitivity C-reactive protein (hsCRP) or vascular endothelial growth factors (VEGF) in the inflammatory stage of Eale’s disease (ED). **A**: The correlation between the serum levels of interleukin-6 (IL-6) and high-sensitivity C-reactive protein (hsCRP) from 41 patients with Eales’ disease (ED) at the inflammatory stage. The IL-6 levels in serum correlated significantly with serum hsCRP levels (Spearman’s correlation coefficient; r=0.4992, p=0.0009). The solid line represents the linear regression curve of the best fit. **B**: The correlation between the serum levels of IL-6 and vascular endothelial growth factor (VEGF) from 41 patients with ED at the inflammatory stage. No significant relation was seen between serum IL-6 levels and serum VEGF levels in the inflammatory stage (Spearman’s correlation coefficient; r=0.08811, p=0.5838). The solid line represents the linear regression curve of the best fit.

Serum concentration of IL-6 significantly correlated with serum hsCRP (Spearman’s correlation coefficient; r=0.4992, p=0.0009) in the inflammatory stage in ED patients, but no statistically significant correlation was seen between serum IL-6 and VEGF (Spearman’s correlation coefficient; r=0.08811, p=0.5838). Furthermore, no significant relation between VEGF and hsCRP (Spearman’s correlation coefficient; r=0.2461, p=0.1208) was seen in the patients in this stage (Figure 1).

No significant relation was found between serum IL-6 and hsCRP (Spearman’s correlation coefficient; r=0.115, p=0.2545) or VEGF (Spearman’s correlation coefficient; r=0.2198, p=0.1469), or between serum VEGF and hsCRP (Spearman’s correlation coefficient; r=0.03568, p=0.8163) in healthy controls.

### Serum levels of interleukin-6, vascular endothelial growth factor, and high-sensitivity C-reactive protein, and vitreous levels of IL-6 and vascular endothelial growth factor in patients with Eales’ disease in the proliferative stage

Serum levels of IL-6 (2.017 pg/ml [1.3–2.98 pg/ml] versus 0.8886 pg/ml [0.46–1.41 pg/ml]; p<0.0001) and VEGF (324.96 pg/ml [132.98–409.78 pg/ml] versus 209.9 pg/ml [32.45–398.78 pg/ml]; p=0.0105) were significantly higher in ED patients in the proliferative stage who had undergone vitrectomy compared to the control patients with macular holes. However, no significant differences were found in serum hsCRP (1.02 mg/l [0.658 −2.357 mg/l] versus 0.9785 mg/l [0.623–1.41 mg/l]; p=0.81) level between these two group (Table 4).

**Table 4:** Demographic and laboratories characteristics of the Eales disease (ED) patients in proliferative stage (those underwent vitrectomy) and controls patients with macular hole underwent vitrectomy surgery

**Characteristics**	**ED patients (proliferative stage’ n=19)**	**Control patients with macular hole (n=16)**	**p value**
Males: Females	18:0	15:1	0.4571
Mean age [years (SD)]	31.8 (8.1)	61.2 (7.5)	0.0001*
Serum IL-6 (pg/ml)	2.017 (0.5476)	0.8886 (0.2589)	<0.0001*
Serum VEGF (pg/ml)	324.96 (91.34)	209.9 (98.475)	0.0105*
Serum hsCRP(mg/l)	1.02(0.685)	0.9785(0.268)	0.81
Vitreous IL-6 (pg/ml)	79.8 (48.53)	12.53 (8.57)	<0.0001*
Vitreous VEGF (pg/ml)	1058.3 (181.5)	16.987 (14.836)	<0.0001*
Vitreous protein (mg/ml)	3.482 (1.83)	0.695 (0.389)	0.0001*
Ratio of IL-6 to protein (pg/mg)	27.39 (12.86)	16.53 (8.65)	0.0071*
Ratio of VEGF to protein (pg/mg)	316.26 (106.32)	26.48 (13.26)	0.0001*

Values are mean (SD) * Significant P value

No significant correlation was found between the serum concentration of IL-6 at this stage of ED and VEGF (Spearman’s correlation coefficient; r=0.0470, p=0.689) or hsCRP (Spearman’s correlation coefficient; r=0.3732, p=0.1544).

Vitreous levels of IL-6 and VEGF were also significantly higher in ED patients in the proliferative stage than in patients with macular holes (79.8 pg/ml [18.65–203.4 pg/ml] versus 12.53 pg/ml [2.32–65.36 pg/ml]; p<0.0001 and 1058.3 pg/ml [750.98–1298.87 pg/ml] versus 16.987 pg/ml [6.98–65.87 pg/ml]; p<0.0001). This difference remained at a significant level when the ratio of IL-6 to vitreous protein (27.39 pg/mg versus 16.53 pg/mg; p=0.0071) and the ratio of VEGF to vitreous protein (316.26 pg/mg versus 26.48 pg/mg; p=0.0001) were considered (Table 4). We calculated the power of statistical testing to establish the null hypothesis in the available vitreous sample used in this study. There was a power of 100% to yield a statistically significant result in this sample size.

**Figure 2:**
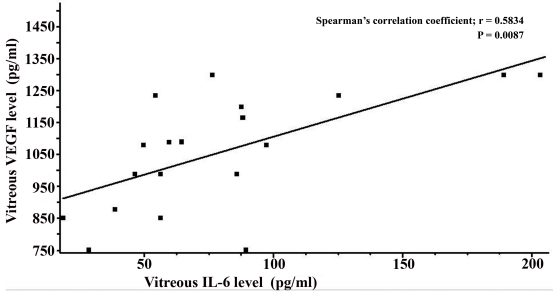
Correlation between vitreous levels of interleukin-6 and vascular endothelial growth factor from 19 patients with Eales’ disease in the proliferative stage. The interleukin-6 (IL-6) levels in vitreous were correlated significantly with vitreous vascular endothelial growth factor (VEGF) levels (Spearman’s correlation coefficient; r=0.5834, p=0.0087). The solid line represents the linear regression curve of the best fit.

There was no significant correlation between the vitreous and serum levels of IL-6 (Spearman’s correlation coefficient; r=0.2789, p=0.2475) and the vitreous and serum levels of VEGF (Spearman’s correlation coefficient; r=0.2045, p=0.4009) in patients in the proliferative stage of ED. Vitreous concentrations of VEGF and IL-6 were significantly higher than that obtained from the serum of ED patients (1058.3 pg/ml versus 324.96 pg/ml; p=0.0001 and 79.8 pg/ml versus 2.017 pg/ml; p<0.0001). However, a statistically significant directly proportional dependence was found between vitreous concentration of IL-6 and VEGF (Spearman’s correlation coefficient; r=0.5834, p=0.0087) in ED patients (Figure 2).

### Association between interleukin-6–174G/C promoter polymorphism and occurrence of Eales’ disease

Allele and genotype frequencies of IL-6**-**174G/C promoter polymorphisms were compared between ED patients (n=121) and healthy controls (n=223); the results are presented in Table 5. No significant departure from the Hardy–Weinberg equilibrium was observed for the IL-6**-**174G/C variant in the ED patients (χ2=3.29; p=0.069) or the healthy controls (χ2=0.357; p=0.549).

**Table 5:** Allele and genotype frequencies of interleukin-6 (IL-6) gene polymorphism in Eales disease (ED) cases and healthy controls

***IL-6* gene variants**	**ED cases (n %)**	**Healthy control (n %)**	**p value**	**OR (95% CL)**
**−174G/C Alleles**				
G	207(85.53)	345(77.35)	0.01008*	1.7314 (1.1362- 2.6386)
C	35(14.46)	101(22.64)		0.5776 (0.379- 0.8802)
**Genotypes**				
GG	91(75.20)	135(60.53)	0.006*	1.9773 (1.2085- 3.2351)
GC	25(20.66)	75(33.63)	0.0114*	0.5139 (0.3054–0.8648)
CC	5(4.13)	13(5.82)	0.49	0.6963 (0.2422–2.0018)

* Significant P- value after Bonferroni correction for multiple testing (i.e., the adjusted p value at the 0.05 significance level is 0.0125)

There was a significant difference in the allelic distribution of IL-6–174G/C polymorphism between the groups (p=0.01008), indicating that the −174G allele (85.53% in ED patients versus 77.35% in controls) may be related to ED occurrence. In addition, the −174C allele (14.46% in ED patients versus 22.64% in controls) was found to be protective against the occurrence of the disease. Individuals with the −174GG genotype were also overrepresented among the patients with ED as compared to controls (75.20% versus 60.53%, p=0.006). On the other hand, −174GC genotype frequency was significantly higher in healthy controls than in the ED patients (20.66% versus 33.63%, p=0.0114). The above associations remained significant after Bonferroni correction for multiple testing (0.0125; significance threshold after correction). We also calculated “power” to test the null hypothesis. Based on the genotype GG homozygote, there is a power of 79.2% to yield a statistically significant result in this sample size.

### Differential expression of interleukin-6, high-sensitivity C-reactive protein, and vascular endothelial growth factor in patients and controls according to interleukin-6–174G/C polymorphism

**Figure 3:**
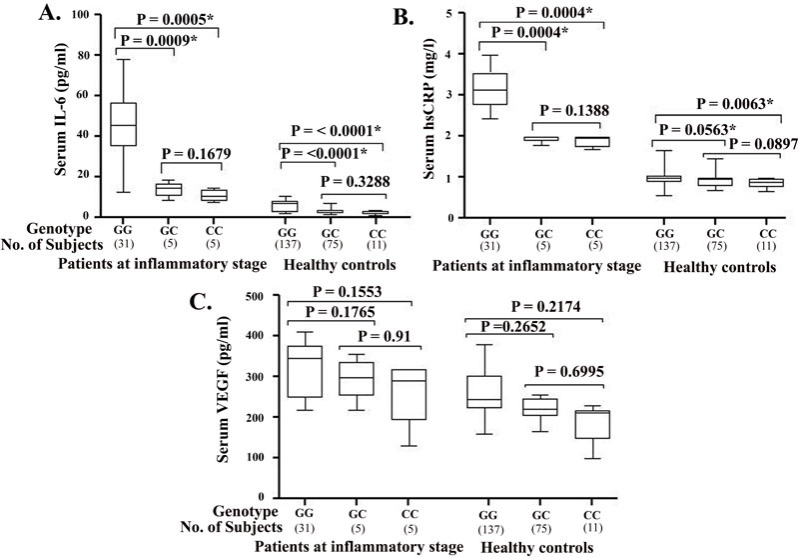
Serum protein concentration in study subjects according to −174G/C genotype. Serum concentrations of: **A**: interleukin-6 (IL-6), **B**: high-sensitivity C-reactive protein (hsCRP), and **C**: vascular endothelial growth factor (VEGF) in patients with Eales’ disease (ED) at the inflammatory stage, as well as the healthy controls, for each −174G/C genotype. The Mann–Whitney U test was performed to determine whether significant differences were present within the same groups of subjects with different −174G/C genotypes. The results of this analysis are indicated in brackets. An asterisk denotes a significant p value.

Figure 3 shows the distribution of serum IL-6, hsCRP, and VEGF concentrations in ED patients in the inflammatory stage and healthy controls in relation to each **-**174G/C polymorphism. Mean serum concentrations of IL-6 were significantly higher in ED patients in the inflammatory stage with the −174GG genotype than those with the −174GC (45.75 pg/ml versus 13.74 pg/ml; p=0.0009) or −174CC genotypes (45.75 pg/ml versus 10.68 pg/ml; p=0.0005). The same trend was also observed in healthy controls. Patients at this stage with the −174GG genotype had significantly higher mean serum concentrations of hsCRP than those with the −174GC (3.172 mg/l versus 1.941 mg/l; p=0.0004) or −174CC genotype (3.172 mg/l versus1.87 mg/l; p=0.0004). Mean hsCRP concentration in healthy controls with the GG genotype was statistically higher than in those with CC genotype (0.9723 mg/l versus 0.8475 mg/l; p=0.0063), but the difference between the hsCRP concentration of controls with the GG genotype and those with the GC genotype was statistically nonsignificant (0.9723 mg/l versus 0.9377 mg/l; p=0.0562). In contrast, the mean serum concentration of VEGF did not differ significantly in patients in the inflammatory stage of ED or healthy controls with different −174G/C genotypes. There were significant differences in the mean serum concentrations of IL-6, hsCRP, and VEGF between inflammatory-stage ED patients and healthy controls for all three IL-6**-**174G/C genotypes (Table 6).

**Table 6:** Mean serum concentrations interleukin-6 (IL-6), high-sensitivity C-reactive protein (hsCRP), and vascular endothelial growth factor (VEGF) in Eales disease (ED) patients at inflammatory stage and healthy controls according to –174G/C genotype

**Genotype**	**Patients (n=41)**	**Controls (n=223)**	**p value**
**IL-6 (pg/ml)**			
GG	45.75 (15.47)	6.026 (2.558)	< 0.0001*
GC	13.74 (3.681)	2.906 (1.383)	0.0002*
CC	10.68 (2.836)	2.344 (0.9589)	0.0022*
**hsCRP (mg/l)**			
GG	3.172 (0.5078)	0.9723 (0.1682)	< 0.0001*
GC	1.941 (0.09016)	0.9377 (0.1615)	0.0002*
CC	1.87 (0.1382)	0.8475 (0.1065)	0.0018*
**VEGF (pg/ml)**			
GG	324.2 (63.23)	251.6 (60.66)	0.0001*
GC	294.9 (50.47)	219.9 (28.7)	0.0335*
CC	270.1 (78.81)	207.1 (45.75)	0.0397*

Values are mean (SD) * Significant P value

**Figure 4:**
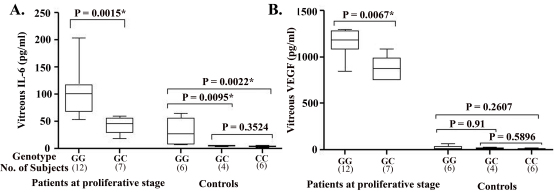
Vitreous protein concentration in study subjects according to −174G/C genotype. Vitreous concentrations of: **A**: interleukin-6 (IL-6) and **B**: vascular endothelial growth factor (VEGF) in patients with Eales’ disease (ED) at the proliferative stage and control patients for each −174G/C genotype. The Mann–Whitney U test was performed to determine whether significant differences were present within the same groups of subjects with different −174G/C genotypes. The results of this analysis are indicated in brackets. An asterisk denotes a significant p value.

In the proliferative stage of ED, vitreous concentrations of IL-6 and VEGF were significantly higher in patients with the GG genotype than the GC genotype (101.5 pg/ml versus 42.58 pg/ml; p=0.0015 and 1151.0 pg/ml versus 899.0 pg/ml; p=0.0067). We found no patients with the CC genotype in this stage. Vitreous IL-6 concentration also showed a similar trend in the case of control patients with macular holes. However, vitreous VEGF concentration in control patients did not show any significant variation with IL-6–174G/C polymorphism (Figure 4) Mean vitreous concentrations of IL-6 and VEGF were significantly higher in patients in the proliferative stage than control patients with macular holes for all IL-6**-**174G/C genotypes (Table 7).

**Table 7:** Mean vitreous concentrations interleukin-6 (IL-6) and vascular endothelial growth factor (VEGF) in Eales disease (ED) patients at proliferative stage and control patients with macular hole according to –174G/C genotype

**Genotype**	**Patients (n=19)**	**Controls (n=16)**	**p value**
**IL-6 (pg/ml)**			
GG	101.5 (48.34)	25.53 (26.59)	0.0023*
GC	42.58 (14.88)	5.415 (0.9661)	0.0061*
CC	-	4.277 (1.263)	-
**VEGF (pg/ml)**			
GG	1151.0 (139.3)	22.91 (22.08)	0.0009*
GC	899.0 (127.8)	16.18 (10.33)	0.0106*
CC	-	11.6 (5.607)	-

Values are mean (SD) * Significant p value.

## Discussion

ED is associated with localized inflammation of the retinal blood vessel walls and is suggested to be an immune-mediated disease. Retinal changes include periphlebitis, peripheral nonperfusion, and neovascularization. At the proliferative or advanced stage of the disease, newly formed retinal vessels become prone to developing vitreous hemorrhage, thus resulting in profound visual loss [4,6]. The impact of this disease is profound because it remains one of the leading causes of ocular morbidity on the Indian subcontinent and affects patients in the most productive period of their life. In light of the evidence linking inflammation and ED, it is of interest to determine whether cytokines such as IL-6 and the proposed involvement of IL-6 gene polymorphism in the in vivo production of this protein illuminate the pathogenesis of this disease.

In this report, we found that the patients in “active” or inflammatory stage have elevated serum IL-6 levels. Whether increased IL-6 merely reflects an acute phase reaction in these patients was investigated by measuring circulating hsCRP levels. CRP is a major acute phase reactant that is primarily regulated by proinflammatory cytokines; it becomes elevated in response to a variety of infections and inflammatory conditions [26,27]. At the same time, CRP levels are rather stable among healthy individuals and reflect the extent of underlying systemic inflammation [28]. The hsCRP assay is highly sensitive compared to measurement of basic CRP, thus allowing patients who have low levels of inflammation to be distinguished [26]. As compared to healthy controls, levels of hsCRP increased more than twofold were found in ED patients in the stage of active perivasculitis. Serum concentrations of IL-6 are known to augment the production of CRP [10], which may also reflect the significant association of these two proteins in ED patients at this stage. In the inflammatory stage, circulating VEGF concentrations were also significantly higher in ED patients compared to healthy controls, but serum VEGF concentrations did not correlate with IL-6 or hsCRP. From these data, it can be interpreted that in the inflammatory stage of the disease, VEGF synthesis does not depend specifically on IL-6, but is rather an associated effect of different proinflammatory cytokines that are secreted as a response to some innate immune stimuli. In line with this explanation, a link between proinflammatory cytokines and VEGF was demonstrated for human peritoneal mesothelial cells in vitro. Incubation of mesothelial cells with interleukin-1α (IL-1α), tumor necrosis factor-α, and thrombin results in a significant increase in VEGF synthesis [29].

During the proliferative stage of ED, inflammation subsided clinically which is also demonstrated by an almost normal level of CRP in the serum of the patients, retinal neovasculation and vitreous hemorrhage have already developed as a consequence of retinal hypoxia and ischemia [14]. At this stage, the vitreous collected from ED patients following vitrectomy is a unique material for analysis of intraocular concentration and/or synthesis of several proteins that orchestrate this pathology. However, the intravitreous increase of a particular protein does not necessarily signal intraocular production; it could reflect only the nonspecific increase of total vitreal proteins observed in these patients, which may be caused by the disruption of the blood–retinal barrier. In this regard, we analyzed the concentration of vitreous proteins that were higher in the ED patients than in control patients with macular holes, and the absolute intravitreous concentration of IL-6 and VEGF were adjusted by the total vitreal protein. In agreement with a previous study [15], we found elevated intravitreous concentrations of IL-6 and VEGF in patients with ED in relation to control patients, not only in absolute terms, but also after adjustment.

IL-6 has several roles in the neovascularization process. It can increase endothelial cell permeability in vitro by rearranging actin filaments and by changing the shape of endothelial cells [30]. Furthermore, IL-6 increases the expression of VEGF [17]. These reports, taken together with our results, suggest that IL-6 may promote vascular permeability along with VEGF in ED. On the other hand, VEGF is a specific mitogen for vascular endothelial cell and therefore has a central role in the physiologic, as well as pathological, angiogenesis [19]. In the current study, vitreous IL-6 level was significantly correlated with vitreous VEGF concentration, thereby also supporting the known ability of IL-6 to act as an indirect inducer of angiogenesis by inducing VEGF expression [17].

The present study did not show any significant correlation between intraocular and serum concentrations of IL-6 and VEGF, and the vitreous concentrations of these factors are significantly higher than the serum concentrations in ED patients. Therefore, these results reinforce the concept that the intraocular synthesis of IL-6 and VEGF, but not serum diffusion [15], is the primary cause of the increased intravitreous concentration of these parameters in ED patients.

Considering the pivotal role that IL-6 plays in the pathogenesis of ED, i.e., its role in modulating acute phase inflammatory response during the inflammatory stage and angiogenic response in the proliferative stage, there is a possibility that differential IL-6 production may influence the susceptibility and severity of ED. It has been established that the constitutive levels of IL-6 are known to be genetically controlled [22], but the putative influences of differential gene expression products of IL-6 on ED onset remain unknown. Within the IL-6 promoter region, one microsatellite and four SNP have been investigated [31]. Among them, a common functional G/C polymorphism located within the 5′ regulatory sequence of the IL-6 gene at position −174 could affect IL-6 expression [22]. In the present study, we found a significant association of the IL-6–174G allele, as well as the −174GG genotype, with the occurrence of ED. In contrast, the heterozygous GC genotype was significantly higher in the controls and gives protection against ED occurrence. Thus, it is suggested that the C allele has a masking effect over the G allele in the heterozygous genotype; this may be due to a complex interaction of both alleles when present codominantly. Under such conditions, it is postulated that the C allele induces a protective response that prevents individuals from developing the disease. However, we did not observe any significant association of the CC genotype with the onset of ED or with a protective influence against development of ED.

In vitro studies revealed that the G allele of the −174 SNP has been associated with an increased transcriptional response against stimulation such as from endotoxin or interleukin-1β [32], whereas studies investigating the role of the −174G/C promoter polymorphism for the circulating IL-6 concentration in vivo have produced conflicting results. According to Fishman et al. [22], unstimulated plasma IL-6 concentrations were associated with the G allele in healthy individuals. However, others have reported that high plasma IL-6 concentrations in patients and healthy individuals were associated with the C allele and CC genotype rather than with the G allele and the GG or GC genotype [33]. Some studies have also indicated that IL-6 −174G/C polymorphism does not significantly affect plasma IL-6 concentrations [34,35]. We found that both ED and healthy controls with the GG genotype had higher mean serum IL-6 concentrations than those with the −174GC or −174CC genotypes.

Interestingly, IL-6 G/C promoter polymorphism has functional significance [22]. The −174G/C polymorphism is contained in a sequence bearing partial nucleotide homology with the Sma- and Mad-related protein 4 (Smad4) binding element. Smad4 is a transcription factor that participates in the signal transduction cascade of transforming growth factor-β and activin to inhibit the expression of proinflammatory molecules [36]. The C allele at the variant position in the consensus-binding element would bind Smad4 more effectively, and hence repress IL-6 transcription, while substitution by a G-allele at this position decreases the binding efficiency by 90% and therefore increases transcription of the IL-6 gene [37]. Thus, the allele G at the −174 position of the IL-6 gene may be a risk factor for ED manifestation associated with constitutively high IL-6 concentration at the initial and advanced stage of this disease.

It was of interest to determine whether −174G/C polymorphism may similarly affect the serum and/or vitreous concentrations of proteins that are regulated by IL-6 in the inflammatory and proliferative stages of ED. We found that the −174GG genotype was associated with increased mean serum concentrations of hsCRP patients in the inflammatory stage of ED. The concentration of hsCRP in serum was also observed to be higher in the −174GG genotype than in the CC genotype in healthy controls in our population, but the difference between hsCRP levels between healthy individuals with the GG genotype and GC genotype was statistically nonsignificant. These results make it plausible that the −174GG genotype facilitates increased IL-6 release, perhaps due to some influence of innate immune response, in the initial stage of ED, which causes the increased release of acute phase inflammatory proteins, i.e., CRP. This possibility is also supported by the observation that serum concentrations of IL-6 and hsCRP in the inflammatory stage of ED patients, but not in controls, correlate with one another. VEGF differed from the CRP in this study because its concentrations were independent of −174G/C polymorphism and not correlated with IL-6 at the inflammatory stage of ED.

On the other hand, intravitreous concentrations of IL-6 and VEGF in patients, those that are representative of the proliferative, i.e., neovascularization stage of ED, are significantly higher in the GG genotype than the GC genotype. To compare, we also genotyped patients with macular holes, those representing the control group for vitreous analysis, and found that vitreous IL-6 levels but not VEGF levels significantly correlate with −174G/C polymorphism of the IL-6 gene. However, it is not significant to evaluate any correlation of the serum level of IL-6 or VEGF and −174G/C polymorphism in the proliferative stage of ED, because in this stage serum concentration does not influence the vitreous levels of IL-6 and VEGF, and the role of vitreous in retinal disorders is a matter of great interest.

In conclusion, it can be said that IL-6 acts as a regulator of the inflammatory as well as the proliferative stage of ED by inducing stage-specific protein expression. IL-6 expression is directly regulated by the allelic distribution of −174 loci and the enhanced level of IL-6 modulates CRP and VEGF concentrations, respectively, depending on the acute inflammatory stimulation at the initial stage and angiogenic stimulation at the advanced stage of ED. To the best of our knowledge, this is the first effort to investigate IL-6 gene polymorphism and its role in the stage-specific modulation of IL-6 and consequently VEGF and CRP in ED-affected patients, and to provide a rationale for targeted therapeutic investigation. The study of other functional polymorphisms in the IL-6 gene will contribute to a better understanding of the pathogenesis of ED, providing information that may have diagnostic and therapeutic value in the future.
